# Delayed P100-Like Latencies in Multiple Sclerosis: A Preliminary Investigation Using Visual Evoked Spread Spectrum Analysis

**DOI:** 10.1371/journal.pone.0146084

**Published:** 2016-01-04

**Authors:** Hanni S. M. Kiiski, Sinéad Ní Riada, Edmund C. Lalor, Nuno R. Gonçalves, Hugh Nolan, Robert Whelan, Róisín Lonergan, Siobhán Kelly, Marie Claire O'Brien, Katie Kinsella, Jessica Bramham, Teresa Burke, Seán Ó Donnchadha, Michael Hutchinson, Niall Tubridy, Richard B. Reilly

**Affiliations:** 1 Neural Engineering Group, Trinity Centre for Bioengineering, Trinity College Dublin, Dublin, Ireland; 2 School of Engineering, Trinity College Dublin, Dublin, Ireland; 3 School of Medicine, Trinity College Dublin, Dublin, Ireland; 4 Department of Neurology, St. Vincent’s University Hospital, Dublin, Ireland; 5 Cognitive and Behavioural Neuroscience Research Group, School of Psychology, UCD College of Human Sciences, University College Dublin, Dublin, Ireland; 6 School of Nursing and Human Sciences, Dublin City University, Dublin, Ireland; Friedrich-Alexander University Erlangen, GERMANY

## Abstract

Conduction along the optic nerve is often slowed in multiple sclerosis (MS). This is typically assessed by measuring the latency of the P100 component of the Visual Evoked Potential (VEP) using electroencephalography. The Visual Evoked Spread Spectrum Analysis (VESPA) method, which involves modulating the contrast of a continuous visual stimulus over time, can produce a visually evoked response analogous to the P100 but with a higher signal-to-noise ratio and potentially higher sensitivity to individual differences in comparison to the VEP. The main objective of the study was to conduct a preliminary investigation into the utility of the VESPA method for probing and monitoring visual dysfunction in multiple sclerosis. The latencies and amplitudes of the P100-like VESPA component were compared between healthy controls and multiple sclerosis patients, and multiple sclerosis subgroups. The P100-like VESPA component activations were examined at baseline and over a 3-year period. The study included 43 multiple sclerosis patients (23 relapsing-remitting MS, 20 secondary-progressive MS) and 42 healthy controls who completed the VESPA at baseline. The follow-up sessions were conducted 12 months after baseline with 24 MS patients (15 relapsing-remitting MS, 9 secondary-progressive MS) and 23 controls, and again at 24 months post-baseline with 19 MS patients (13 relapsing-remitting MS, 6 secondary-progressive MS) and 14 controls. The results showed P100-like VESPA latencies to be delayed in multiple sclerosis compared to healthy controls over the 24-month period. Secondary-progressive MS patients had most pronounced delay in P100-like VESPA latency relative to relapsing-remitting MS and controls. There were no longitudinal P100-like VESPA response differences. These findings suggest that the VESPA method is a reproducible electrophysiological method that may have potential utility in the assessment of visual dysfunction in multiple sclerosis.

## Introduction

Visual dysfunction is a common feature of multiple sclerosis (MS). MS is the most common chronic inflammatory demyelinating disease affecting the central nervous system of young adults in Western countries leading to severe disability with no cure [1]. The visual system of most MS patients is affected during the course of the condition, which can eventually lead to disability along with the degradation of other central functions in motor and sensory systems. Approximately 95% of MS cases present with optic neuritis (ON) causing patients to experience a decline in vision over a 7–10 day period with vision improving within 30 days of onset [2]. Other visual dysfunctions in MS include nystagmus, internuclear ophthalmoplegia and gaze palsies [3]. In clinical practice visual function in MS patients is often evaluated with measures of visual acuity, color vision and visual field [4, 5], however, these do not provide information on the brain processes underlying potential dysfunction.

Visual Evoked Potentials (VEPs) are visually evoked electrophysiological signals extracted from the electroencephalographic (EEG) activity recorded from the scalp [6]. VEPs have been shown to depend on the functionality of the patient’s central vision at any level of the visual pathway (the eye, retina, optic nerve, optic radiations and occipital cortex) [6]. The VEP method is utilised in clinical practise as part of the evaluation of the optic nerve in order to determine visual defects such as ON [7]. The VEP has been deemed to be a valuable analysis tool in MS and it may, in combination with other modalities, be utilized as a prognostic marker when monitoring MS disease progression [6, 8–13].

Prior studies [8, 11–13] examining VEPs in MS found the P100 responses of MS patients to have prolonged latencies and reduced amplitudes, and that these properties related to motor and visual dysfunction. A similar pattern of the P100 responses was reported in moderately and severely fatigued MS subjects in a recent study [14]. VEPs have also been reported to predict MS disability [12, 13]. These studies indicate that the VEP can be a highly useful tool to assess the optic nerve and monitor disease progression in MS.

The Visual Evoked Spread Spectrum Analysis (VESPA) method has been proposed as an alternative approach to measuring visual processing using EEG [15, 16]. Unlike the VEP, which uses discrete visual stimulus events, the VESPA method involves a *continuous* visual stimulus modulating over time, which is presented to the subject. One specific approach is to modulate the luminance of the stimulus according to a stochastic waveform, with a high refresh rate (typically 60 Hz). This stochastic waveform has its power broadly spread over a range of frequencies, as opposed to being a simple repetitive stimulus at a single frequency. The VESPA and VEP measures are found to be similar with their temporal profiles being highly correlated over occipital scalp. But their distribution on the scalp is markedly different with the VESPA being much less broadly distributed suggesting contributions from a more restricted area of cortex [15].

The VESPA method allows for the rapid acquisition of a visual evoked response with a detailed temporal profile, including a P100-like component, and a high signal-to-noise ratio [15]. Furthermore, a major disadvantage of the VEP method is that the EEG must be averaged over many trials to obtain a stable VEP response—typically a minimum of 64 trials with 200–400 trials being preferable [6, 17]. It has also been suggested that the VESPA may be more sensitive to individual subject differences relative to VEPs because it interrogates the visual system across a range of stimuli rather than just with extreme discrete events [15]. VESPA responses can also be acquired to each of several simultaneously presented stimuli, something that is not possible with the VEP. Indeed, the notion of rapidly obtaining distinct responses to multiple simultaneously presented stimuli across the visual field is something that has already been investigated using an approach that represents a special case of the VESPA [18].

To our knowledge, this is the first study examining the VESPA method with MS patients. Based on earlier cross-sectional VEP and VESPA studies [8, 11–13, 15, 16] it was hypothesized that 1) MS patients would have prolonged latencies and reduced amplitudes of the P100-like VESPA component relative to healthy controls, 2) secondary-progressive MS (SPMS) subgroup would have prolonged latencies and reduced amplitudes of the P100-like VESPA component relative to relapsing-remitting MS (RRMS) patients and controls, that 3) over 3-year period the latencies of the P100-like VESPA component would be increasingly delayed and the amplitudes of the P100-like VESPA component increasingly reduced in MS patients, especially in SPMS subgroup, compared to the controls and RRMS subgroup due to increase in pathological brain changes. Therefore, the main aim of the study was to provide a preliminary investigation into the utility of the VESPA method for probing and monitoring visual dysfunction in MS. The findings indicate VESPA to be a reproducible electrophysiological method that may have potential utility in assessing visual dysfunction and disease progression in multiple sclerosis.

## Materials and Methods

### Subjects

The data were collected as part of a larger research project examining cognitive function in MS [19–25]. Fifty-six MS patients satisfying the revised McDonald criteria for MS [7, 26] were recruited in collaboration with the Department of Neurology in St. Vincent’s University Hospital, Dublin Ireland. Exclusion criteria included a current use of benzodiazepines or neuroleptics with a minimum suspension period of seven days, a history of alcohol or drug misuse, head injury or stroke. In addition, 53 age-matched healthy controls were recruited. The subjects were asked to participate in the study once a year over a period of three consecutive years. The mean time between the baseline and Month 12 sessions was 374.35 days (*SD* = 56.96), and 391.42 days (*SD* = 45.35 days) between Month 12 and Month 24. Ethical approval was obtained from the Ethics and Medical Research Committee of the St. Vincent’s Healthcare Group and the research was conducted according to the principles expressed in the Declaration of Helsinki. Written informed consent was obtained from all subjects on each testing occasion.

First the quality of EEG data was examined. Noisy data due to bad electrode contact or excessive motor activity was identified by visual inspection and excluded from the study. Following this, a total of 43 MS patients (mean age = 42.91 years) and 42 age-matched healthy controls (mean age = 40.9 years) were included in the study at Month 0. The MS sample included 23 relapsing-remitting MS (RRMS) and 20 secondary-progressive MS (SPMS) patients. The two follow-up sessions were conducted 12 months post-baseline (i.e. Month 12) with 24 MS patients (15 RRMS and 9 SPMS) and 23 healthy controls, and again at Month 24 with 19 MS patients (13 RRMS, 6 SPMS) and 14 healthy controls. 19 MS patients had a clinical history of optic neuropathy and 24 MS patients had no history of optic neuropathy. Most subjects completed two VESPA runs per testing session, which were subsequently averaged to generate a single VESPA response for each year. Some subjects did not complete a second VESPA run due to time limitations (Month 0 N = 18, Month 12 N = 4, Month 24 N = 3), and therefore the VESPA response from their single trial was utilised in the subsequent analyses. MS patients also completed a physical examination each year, including Kurtzke Expanded Disability Status Scale (EDSS) [27] and Snellen visual acuity test at baseline [28], which were administered by a neurology registrar in the Department of Neurology. Snellen visual acuity data were converted to decimal values [29]: a vision of 20/20 converted in decimal form to 1.0, 20/25 to 0.8, 20/40 to 0.5, 20/100 to 0.2, etc. Two MS patients had a relapse between baseline and 12 months, and one between Month 12 and Month 24. 7 MS patients had one or more relapses 12 months before baseline, and 5 MS patients between 12 and 24 months prior the baseline. Table 1 displays the demographic and behavioural data of the subjects. Table 2 shows the average disease duration and drug therapies used by MS patients in each year.

**Table 1:** Demographical and behavioural data of MS patients and controls.

	Male / female	Normal VEP / abnormal VEP	ON / no ON	Age (mean, SD)	Edu years (mean, SD)	VA (mean, SD)	EDSS (M, IQR)	Rel.*
**MS patients (N = 43)**								
Month 0	23 / 20	3 / 7	19 / 24	42.91, 10.03	15.31, 3.4	0.84, 0.26	3, 4.5	2
Month 12	12 / 12	1 / 4	10 / 13	41.98, 10.72	14.46, 3.45	N/A	2, 4.5	1
Month 24	9 / 10	1 / 4	9 / 9	42.88, 11.31	14.26, 3.49	N/A	2, 4.5	0
**RRMS (N = 23)**								
Month 0	10 / 13	0 / 4	8 / 15	37.84, 8.61	15.78, 3.34	0.92, 0.21	2, 1.5	2
Month 12	5 /10	0 / 3	4 / 10	37.81, 9.95	14.67, 3.62	N/A	1.5, 2	1
Month 24	5 / 8	0 / 3	4 / 8	38.39, 10.48	14.31, 3.75	N/A	2, 1.5	0
**SPMS (N = 20)**								
Month 0	13 / 7	3 / 3	11 / 9	48.74, 8.34	14.74, 3.48	0.72, 0.3	6.5, 1.4	0
Month 12	7 / 2	1 / 1	6 / 3	48.94, 8.35	14.11, 3.33	N/A	6, 0.5	0
Month 24	4 / 2	1 / 1	5 / 1	52.6, 5.59	14.17, 3.19	N/A	6.3, 1.5	0
**Controls (N = 42)**								
Month 0	26 / 16	N/A	N/A	40.9, 9.11	1.22, 3.1	N/A	N/A	N/A
Month 12	16 / 7	N/A	N/A	43.82, 11.22	17.6, 3.3	N/A	N/A	N/A
Month 24	10 / 4	N/A	N/A	45.39, 11.08	17.75, 3.08	N/A	N/A	N/A

Note. *M* = median, *IQR* = interquartile range, RRMS = relapsing-remitting MS patients, SPMS = secondary-progressive MS patients, ON = history of optic neuritis, Edu years = years of education, VA = Visual acuity expressed as decimal values and averaged from both eyes, EDSS = Expanded Disability Status Scale, Rel.

* = number of MS patients who had a relapse between Month 0 and Month 12 (Month 0 column), between Month 12 and Month 24 (Month 12 column).

**Table 2:** Disease duration and drug treatment data of MS patients.

	Disdur_S (mean, SD)	Disdur_dx (mean, SD)	N Interferon β-1a	N Interferon β-1b	N Natalizumab	N Clinical trial	N No current treatment
**MS patients (N = 43)**							
Month 0	15.74, 9.88	11.3, 7.73	10	7	5	1	10
Month 12	15.38, 11.73	10.28, 9.39	7	7	5	1	4
Month 24	15.25, 12.77	9.72, 9.61	6	5	5	1	2
**RRMS (N = 23)**							
Month 0	8.96, 6	5.96, 4.94	6	5	5	1	0
Month 12	8.07, 4.99	4.34, 2.73	4	5	5	1	0
Month 24	7.86, 5.25	4.16, 2.85	3	4	5	1	0
**SPMS (N = 20)**							
Month 0	23.59, 7.32	17.48, 5.39	4	2	0	0	10
Month 12	27.5, 9.31	20.17, 8.01	3	2	0	0	4
Month 24	31.27, 8.44	21.77, 7.65	3	1	0	0	2

Note. RRMS = relapsing-remitting MS patients, SPMS = secondary-progressive MS patients, Disdur_S = years since the first symptom, Disdur_dx = years since the MS diagnosis

### VESPA stimuli

**Fig 1:**
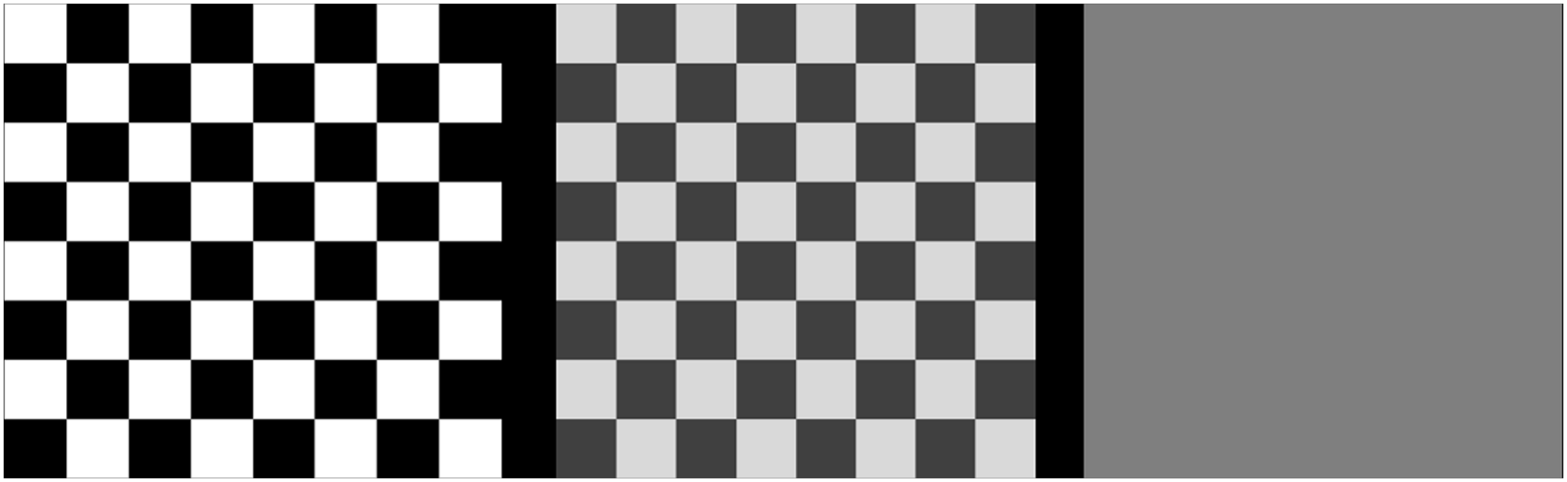
64 square mean luminance checkerboard patterns with varying contrast.

The binocular VESPA stimulus presented to the subjects of this study consisted of a checkerboard with 64 squares, as shown in Fig 1. The contrast of this checkerboard could vary over 68 levels between 0 and 100% with the mean luminance of each of these 68 levels being approximately equal. On every refresh of a monitor set to 60Hz, the contrast of the checkerboard was modulated by a stochastic signal. The VESPA response on each electrode channel was then derived under the assumption that the recorded EEG represented a convolution of that stochastic signal with an unknown impulse response plus noise, i.e.,

y(t)=w(τ)*x(t)+noise

where y(t) is the EEG, x(t) is the known stochastic modulation signal, * indicates convolution and the noise is assumed to be Gaussian. w(τ) is the VESPA; that is, the impulse response function to the contrast of the stimulus [15]. This was estimated using MATLAB [30].

The power of the waveforms used to modulate the luminance of the visual stimulus is spread over a range of frequencies and as a result they are termed spread spectrum waveforms. The VESPA method allows for the presentation of simultaneous stimuli that produce separate VESPA responses [15].

### Experimental procedure, EEG acquisition and data analysis

Subjects were seated 70 cm from a cathode ray tube (CRT) computer monitor with a screen resolution of 1024 x 768 pixels, and at a refresh rate of 60Hz. Each stimulus subtended a visual angle of approximately 5° degrees vertically and horizontally. Stimulation was performed binocularly. Subjects were asked to maintain their focus on the presented stimuli. Each subject typically underwent two VESPA runs per session, and each run lasted 120 seconds. Where possible, the VESPA from two runs were later averaged for data analysis.

EEG data were recorded using the ActiveTwo Biosemi^™^ system in a soundproofed, darkened room from 134 electrodes (128 scalp electrodes), and organized according to the 10–5 system [31]. The vertical and horizontal electro-oculograms were recorded bilaterally from approximately 3 cm below the eye and from the outer canthi respectively. EEG data were filtered over the range 0–134 Hz and digitized at 512 Hz. Subsequently, the EEG was digitally filtered with a high-pass filter with passband above 0.5 Hz and -60 dB response at 1 Hz and a low-pass filter with 0–35 Hz passband and -50 dB response at 45 Hz. Data on channels identified as noisy were replaced by interpolating the data recorded at six neighbouring sites.

The VESPA impulse response was estimated using the method of linear least squares based on the known stimulus signal and the measured EEG. VESPA responses at Oz were plotted as a function of time for each subject and for each year. The decision to focus the analysis on channel Oz, despite having access to 128 channels of scalp data was based on previous studies showing the VESPA to be unimodally distributed around Oz. Once the VESPA responses had been plotted for each of the subjects, the values for the P100-like VESPA component peak amplitude and peak latency were extracted manually. These were then subjected to statistical analyses.

Statistical analyses on demographical and behavioural data were completed using IBM SPSS Statistics software [32]. For cross-sectional analyses t-test and one-way ANOVA were utilised to compare the groups. Longitudinal analyses were based on mixed-design ANOVA with group as a between-subject factor and time (i.e. test session) as a within-subject factor. The binocular VESPA response (latency, amplitude) was the main dependent variable. The Spearman correlation coefficient was applied as it does not use actual data values, but rather a rank-order correlation coefficient that measures association at the ordinal level. Pearson correlation coefficients were used to assess between-session (Month 0 vs. Month 12) test–retest reliability [33]. Sensitivity to change in P100-like VESPA amplitude and latency between session (Month 0 vs. Month 12) in MS patients was estimated with the standardized response mean (SRM = mean change / standard deviation of change; where mean change is computed by subtracting amplitude/latency value at Month 12 from amplitude/latency value at Month 0) and partial correlation using relapse rate (number of relapses 24 months prior the baseline) as a covariate.

## Results

### Cross-sectional differences between the MS patients and the healthy controls in P100-like VESPA component

**Fig 2:**
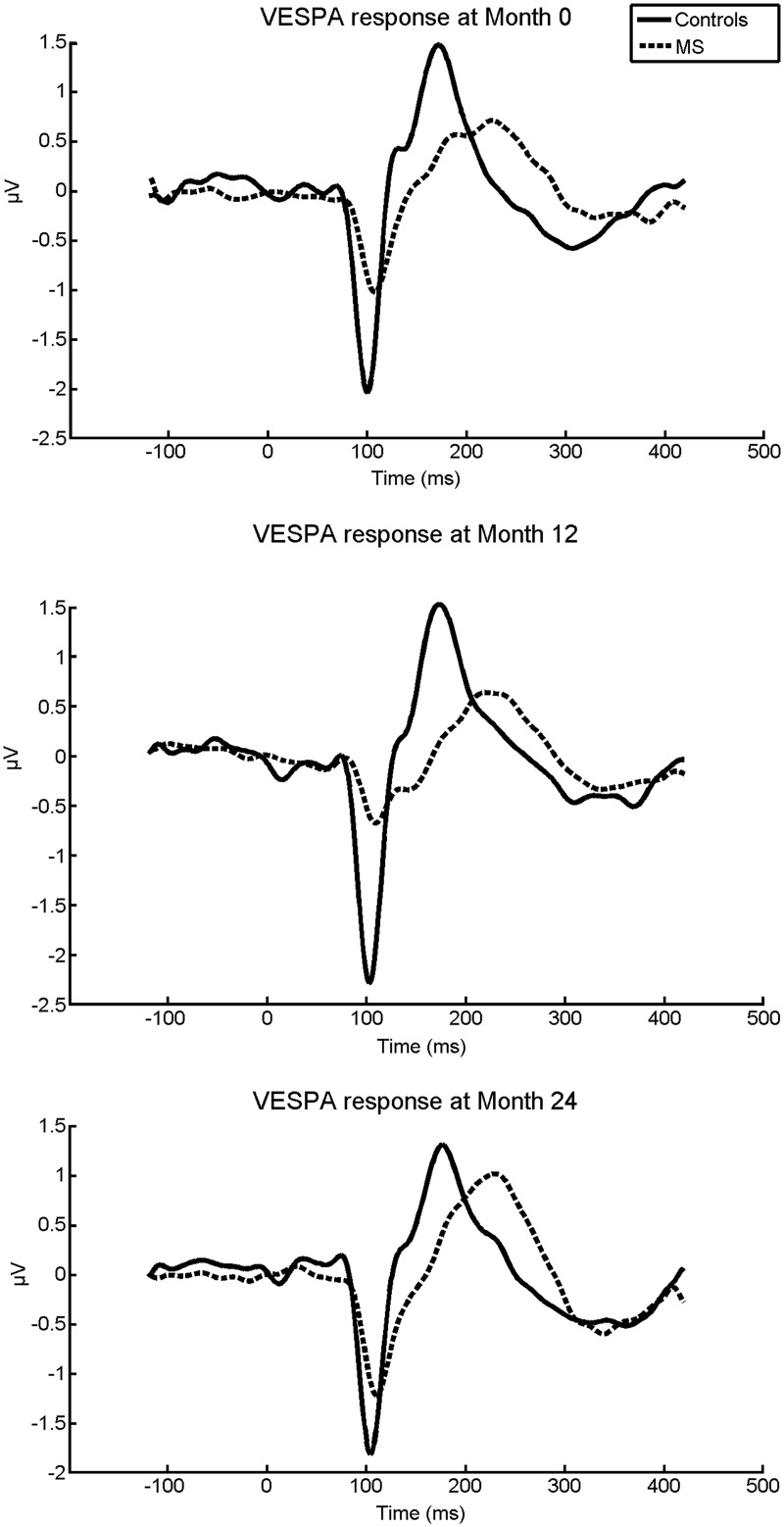
Averaged VESPA responses of MS patients and controls at Month 0, Month 12 and Month 24.

**Fig 3:**
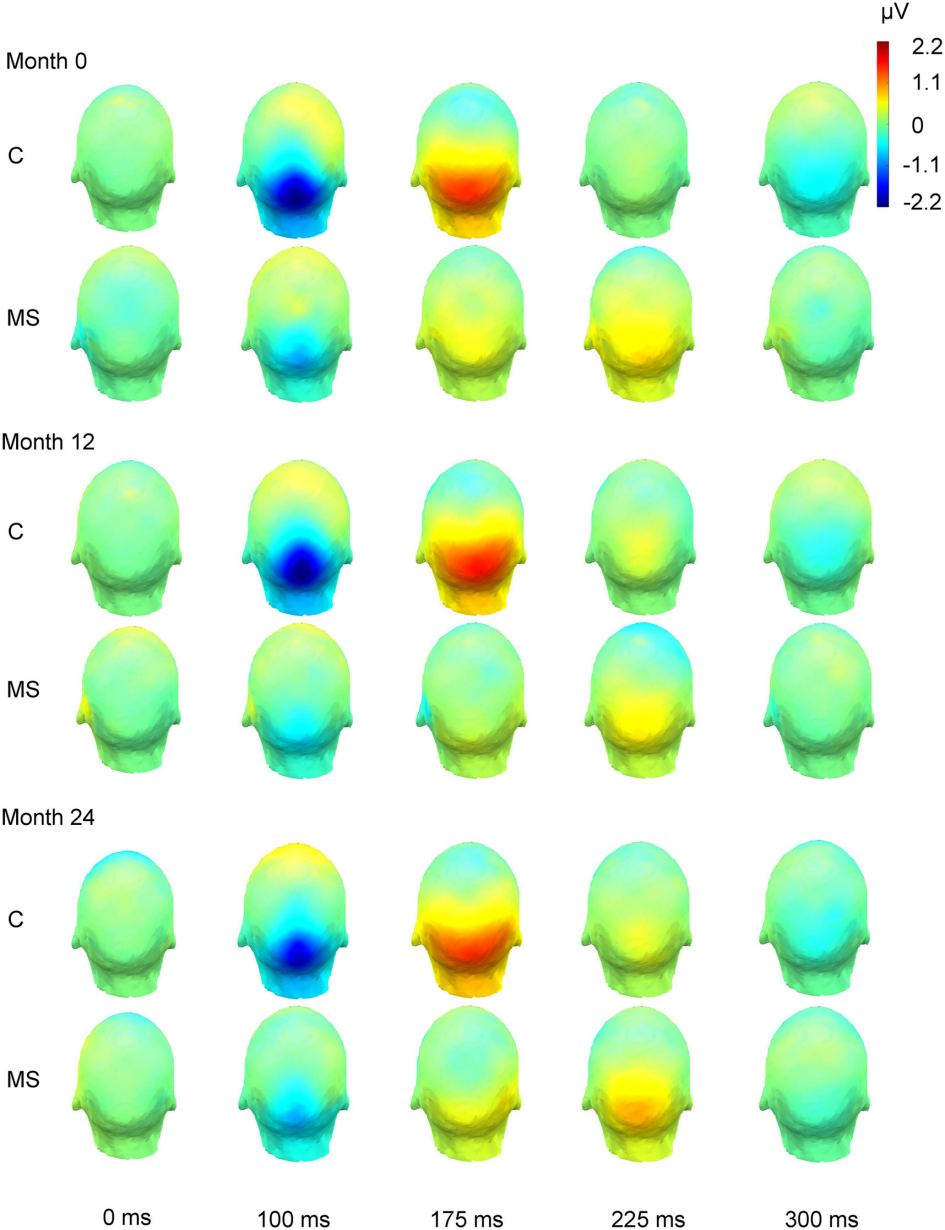
Activation over the scalp during averaged VESPA responses of healthy controls and MS patients.

At baseline MS patients showed significantly delayed P100-like VESPA latencies (*t*(72) = 3.37, *p* = 0.001) compared to healthy controls. There was a trend (*p* = 0.07) indicating that MS patients had reduced amplitudes relative to healthy controls. The findings are displayed in Figs 2 and 3, and in Table 3. Results remained the same when MS patients with relapses within 24 months pre- and post-baseline were excluded from the analysis.

**Table 3:** VESPA-like P100 latencies and amplitudes for controls, all MS patients, RRMS patients, SPMS patients, and MS patients with and without a history of optic neuropathy.

	MS (*Mean*, *SD*)	RRMS (*Mean*, *SD*)	SPMS (*Mean*, *SD*)	C (*Mean*, *SD*)	ON *(Mean*, *SD)*	noON *(Mean*, *SD)*
**P100-like VESPA latency**						
Month 0	187.39, 43.19	176.35, 41.2	200.1, 42.9	160.94, 27.80	188.81, 45.19	186.25, 42.48
Month 12	192.81, 49.87	184.89, 42.3	206.0, 60.9	167.45, 34.06	209.58, 54.07	180.84, 44.77
Month 24	195.98, 45.53	192.00, 43.3	204.6, 53.3	171.88, 29.05	204.08, 46.27	188.70, 46.03
**P100-like VESPA amplitude**						
Month 0	1.91, 1.22	1.87, 1.09	1.95, 1.37	2.38, 1.19	1.89, 0.83	1.92, 1.47
Month 12	1.80, 0.88	1.96, 0.99	1.53, 0.61	2.42, 1.22	1.87, 0.82	1.75, 0.95
Month 24	1.81, 1.26	2.11, 1.29	1.16, 0.98	2.22, 1.11	1.83, 1.21	1.79, 1.37

Note. SD = standard deviation, RRMS = relapsing-remitting MS patients, SPMS = secondary-progressive MS patients, C = controls, ON = MS patients with a history of optic neuropathy, noON = MS patients without a history of optic neuropathy.

### Cross-sectional differences between the RRMS patients, SPMS patients and the healthy controls in P100-like VESPA component

**Fig 4:**
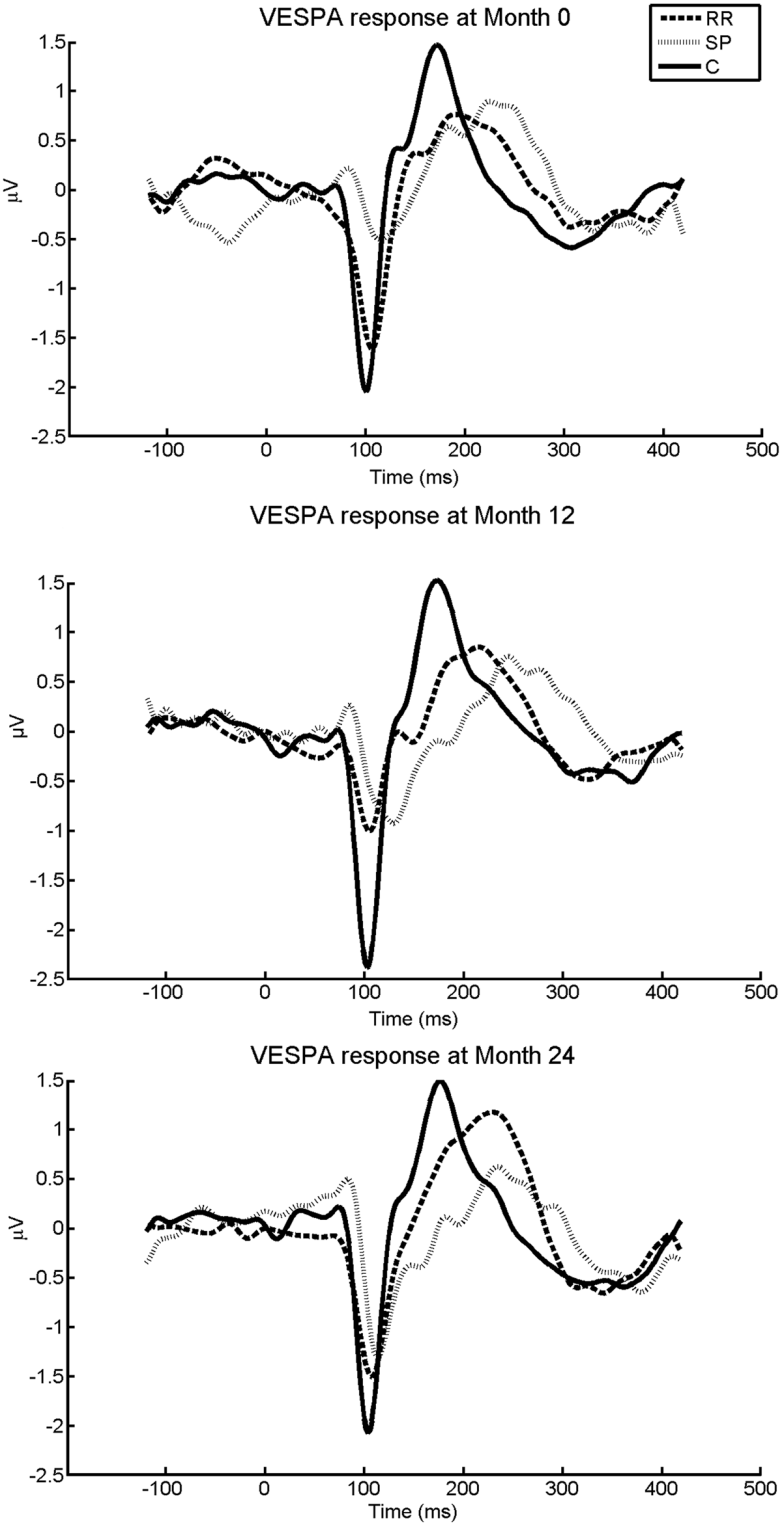
Averaged VESPA responses of relapsing-remitting MS patients (RR), secondary-progressive MS patients (SP) and controls at Month 0, Month 12 and Month 24.

RRMS, SPMS and healthy controls differed significantly in P100-like VESPA latency (*F*(2,82) = 8.24, *p* = 0.001). Tukey post-hoc tests showed that the SPMS patients had the most delayed P100-like VESPA latencies in comparison to the healthy controls (see Fig 4, and Table 3. There were no statistically significant differences in amplitude. Furthermore, when MS patients with relapses within 24 months pre- and post-baseline were excluded from the analysis the results remained the same.

### Cross-sectional differences between the MS patients with and without optic neuropathy in P100-like VESPA component

MS patients with and without a history of optic neuropathy did not differ in P100-like VESPA amplitudes (*p* > .05) and latencies (*p* > .05). (Table 3).

### Correlation between visual acuity and P100-like VESPA component

Visual acuity did not correlate with P100-like VESPA amplitudes or latencies (*p* > .05).

### Longitudinal differences between the multiple sclerosis patients and the healthy controls in P100-like VESPA component

MS patients had more prolonged P100-like VESPA latencies compared to the healthy controls over the 24 Month period (group main effect, *F*(1,24) = 5.81, *p* = 0.02), after MS patients with relapses within 24 months were excluded from the analysis. There were no other statistically significant time or interaction effects over the 24-month period. When the groups were compared at each timepoint separately, P100-like VESPA latencies were significantly prolonged in MS patients relative to the healthy controls at Month 0 and Month 12, and P100-like VESPA amplitudes were reduced in MS patients at Month 12. After MS patients with relapses within 24 months were excluded from the analysis, also the P100-like VESPA amplitudes were observed to be reduced in MS patients at Month 24 (Figs 2 and 3, Table 3).

### Longitudinal differences between the RRMS patients, SPMS patients and the healthy controls in P100-like VESPA component

No significant group, time or interaction effects were found between the RRMS patients, SPMS patients and healthy controls in the P100-like VESPA variables over the 24-month period (*p* < 0.05). There were no significant differences when the groups were compared at each timepoint separately (*p* > 0.05). After controlling for relapses, P100-like VESPA latency were delayed in RRMS patients relative to controls (*p* < 0.05) (Fig 4, Table 3).

### Longitudinal differences between the MS patients with and without optic neuropathy in P100-like VESPA component

No significant group, time or interaction effects were found between the MS patients with and without a history of optic neuropathy in the P100-like VESPA variables over the 24-month period (*p* > 0.05). (Table 3).

### VESPA method: test-retest reliability, sensitivity to change and test performance

The test-retest reliability was measured with healthy controls who had data at baseline and at Month 12. The test-retest reliability was high for P100-like VESPA latency (*r*_*Pearson*_ = .87, *p* < .001; *ICC*_*average measures*_ = .93, *95% CI* .82-.97; *Cronbach’s α*_*Standardized*_ = .93) and moderately high for P100-like VESPA amplitude (*r*_*Pearson*_ = .67, *p* = .001; *ICC*_*average measures*_ = .80, *95% CI* .51-.92; *Cronbach’s α*_*Standardized*_ = .80). Sensitivity to change of the P100-like VESPA response in MS patients was minor (amplitude: *SRM* = .25; latency: *SRM* = -.14).

**Fig 5:**
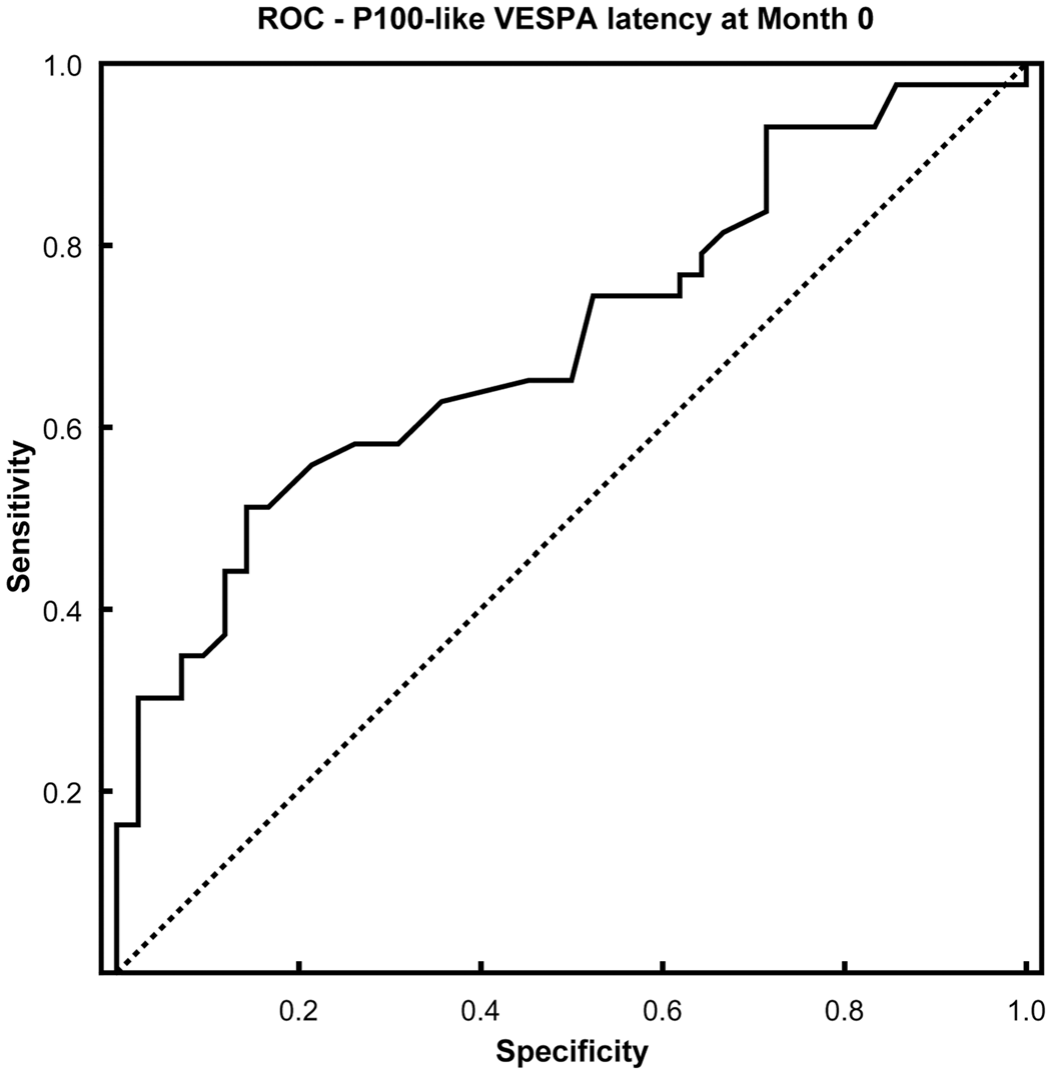
Discrimination performance (ROC curve) of P100-like VESPA latency for MS and controls at Month 0.

**Fig 6:**
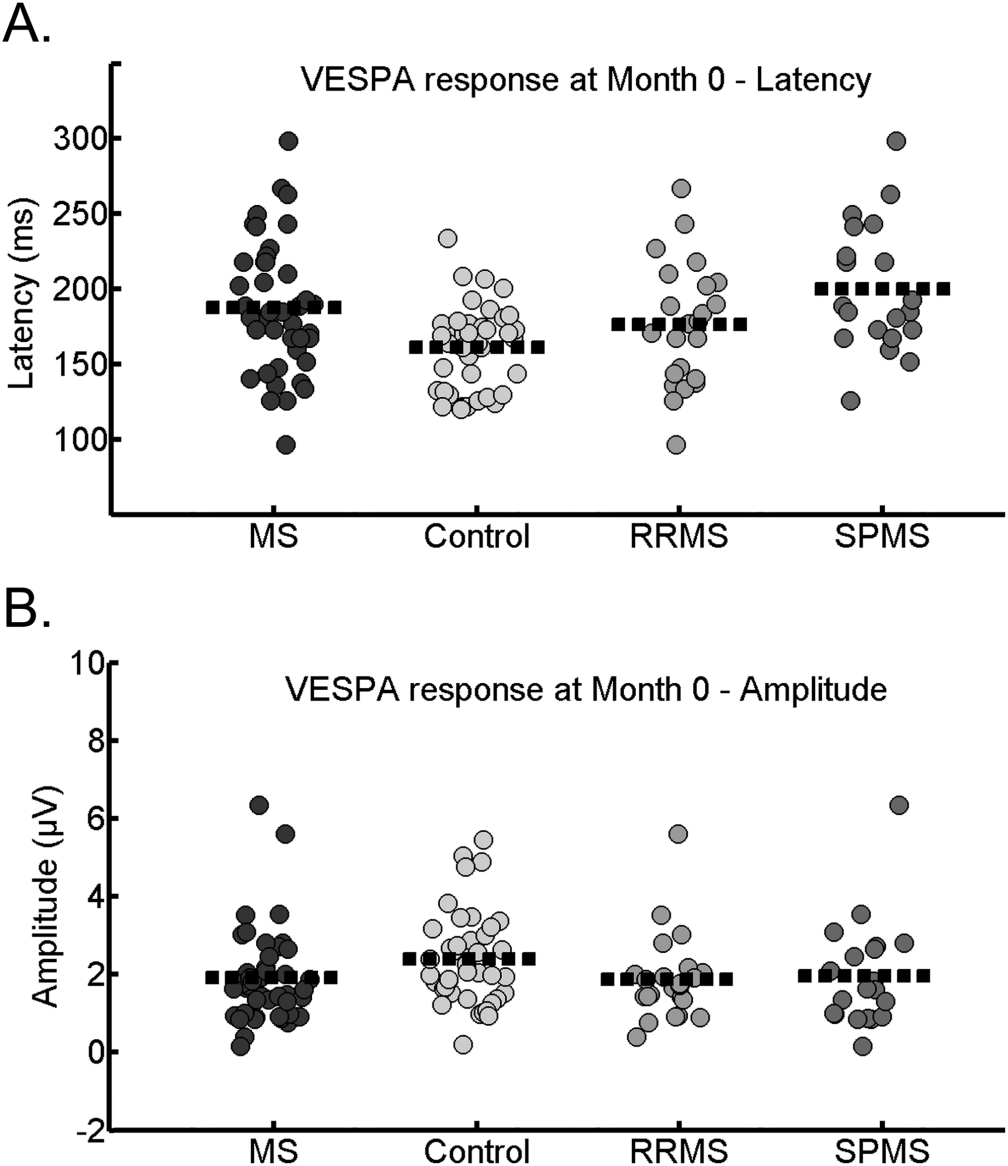
A. Scatter plot of P100-like VESPA latency (A) and amplitude (B) in controls and MS, relapsing-remitting MS patients (RRMS) and secondary-progressive MS patients (SPMS) at Month 0 (mean latency and mean amplitude in each group indicated with a dotted line).

The sensitivity and specificity of P100-like VESPA latency was moderate, but it performed significantly better than a random test in distinguishing between MS patients and healthy controls at Month 0 (*ROC* = .70, *SE* = .057, *95% CI* [.58, .81], *p* < .01; see Figs 5 and 6A, and S1 Table). P100-like VESPA amplitude did not successfully discriminate between the groups (*p* > .05, Fig 6B).

## Discussion

### The results of the present study

The utility of the VESPA method in measuring visual dysfunction in MS has not been investigated prior to this study. However, previous studies have found VESPA and VEP methods to produce similar, but not identical, responses [15], indicating VESPA may be a useful tool to probe and monitor visual dysfunction in MS. The VESPA also allows for the rapid acquisition of a visual evoked response with a complete temporal profile, it has high signal-to-noise ratio, and is plausibly more sensitive to individual subject differences than VEPs [15]. In fact, consistent with the hypotheses, MS patients had significantly prolonged latencies of the P100-like VESPA component in comparison to responses of healthy controls at baseline. SPMS patients had the most delayed latencies of the P100-like VESPA component compared to healthy controls. It is likely that these differences result from a reduction in visual function due to the effects of demyelination, which is more widely spread in the brains of SPMS patients. The SPMS patients deteriorate progressively and most of them experience continuous severe disease related symptoms including visual dysfunction, fatigue and muscular problems. However, the P100-like VESPA activations for the MS patients with and without a history of optic neuropathy did not differ. This may be due to the great variability among the MS patients in the time since last occurrence of optic neuritis (range = 0–45 years). In the longitudinal analysis, the MS patients had prolonged VESPA-like latencies in comparison to healthy controls over the full 24-month period. However, there was no consistent pattern of changes in the P100-like VESPA components over the 24-month period in either group.

### Comparison with prior studies

The results of the present VESPA study are similar to the VEP findings of Balnyte et al. [8], which was expected based on previous studies showing the temporal profiles of VESPA and VEP to be highly correlated and reproducible [15]. They reported MS patients to have greater VEP P100 latency differences and reduced VEP P100 amplitude differences compared to healthy controls. Although the results of the previous VEP studies are not directly comparable in the strictest sense to the current VESPA study due to the differences in the methods, nevertheless, they both produce P100 component. The previous studies also highlight the utility of VEP as a tool for evaluating the function of the optic nerve and its role as a prognostic marker and in MS progression monitoring [8–13]. Moreover, the present results are promising as the VEPs have been shown to be a sensitive measure at detecting abnormalities, including clinically silent lesions [34]. In the present preliminary study the VESPA method was shown to be able to discriminate between MS patients and healthy controls as MS patients had prolonged latencies of the P100-like VESPA component relative to healthy controls.

The clear differences between the groups at Month 0 and the follow-up sessions in latency of the P100-like VESPA component, and the non-existent longitudinal differences, highlight the reproducibility of the P100-like VESPA response, also confirmed by the findings of Lalor et al. [15]. This reproducibility was also highlighted in the present study as test-retest reliability was high, especially for P100-like VESPA evaluation in healthy controls. Reproducibility is an important feature when choosing a reliable tool for clinical practice as it allows for meaningful comparisons to be made between separate responses. Moreover, the lack of longitudinal differences in MS is most likely due to no significant changes in the pathological processes affecting the visual system. Furthermore, the role of remyelination has been previously suggested to be one of the reasons of no visible prolongation effects of VEP P100 latencies over time, or even the shortening of P100 latencies in the VEP follow-up sessions [11], which may be an alternative explanation to the lack of P100-like VESPA component changes over time in MS. This view was supported by our findings, as in RRMS patients the amplitude of the P100-like VESPA component increased at Month 24 relative to the previous years (see Table 3, Fig 4). No relapses were reported in the RRMS patients during the 24 Month period which indicates their disease progression to have remained relatively stable. Taken together, this evidence suggests that there may be restoration of visual function in RRMS. However, the lack of statistically significant longitudinal results may be caused by small sample sizes at Month 24 (e.g. at Month 24 only 6 SPMS patients were included in the analysis), leading to a reduction in the statistical power.

The present study avoided many shortcomings of the prior studies in the area, such as the inclusion of neurological patients as controls [8, 11], the lack of comparison between MS subtypes [8], and that the study groups were observed over unequal amounts of time [11]. Furthermore, in the study of Balnyte et al. [8] about one third of the MS patients examined in the study had a history of ON and this may have been a contributing factor to the high level of VEP abnormalities. In addition, some researchers have theorized that the VEP P100 may contain contributions from ongoing oscillations that are “phase reset” by the discrete presentations of each stimulus [35]. This idea suggests that averaging across multiple trials can produce what appears to be an evoked response simply because the ongoing oscillations following each phase reset are (briefly) temporally aligned. While, few researchers suggest that such a mechanism explains the generation of the VEP in its entirety, any potential contributions it makes to the VEP complicate clinical interpretation in patients with MS. This is particularly true as it is unknown how demyelination would differentially affect evoked and ongoing oscillatory activity in visual cortex. Based on the assumptions underlying the VESPA analysis, it has been suggested that phase-reset contributions to the VESPA are overwhelmingly unlikely [15].

The present study had a number of its own limitations. Most importantly, the conventional VEP was not part of the experimental procedure in the present study, and thus the findings of the present study are exploratory in nature. Therefore, the clinical utility of the VESPA method in detecting visual dysfunction will need to be examined with more comprehensive studies comparing the two methods. These studies should also compare the topographical distribution of VEP and VESPA activations over the scalp as previous studies have suggested VESPA to represent contributions from a more restricted region of cortex than the VEP, potentially due to being generated predominantly by early visual cortical areas [36–37]. If similar results are found in MS patients this may indicate that VESPA response may be more sensitive to the level of demyelination occurring in the white matter tracts of the visual system compared to the conventionally measured VEP P100 which comprises contributions from a number of cortical generators [38–49]. Furthermore, the present study did not test the sensitivity of the VESPA method to specific brain pathways. Previous studies have reported the high-contrast VESPA stimuli to preferentially target the parvocellular pathway within the brain [16, 50], which is thought central for visual acuity and the processing of higher-order patterns, chromatic stimuli and higher spatial frequencies [51]. The parvocellular pathway accounts for approximately 80% of optic nerve fibers, and it consists of thinly myelinated ganglion cells likely to be prone to disruptions caused by widespread demyelination in MS [51]. Thus, comparing the VEP and VESPA methods with respect to brain pathways would be of great value.

Another major limitation of the present study was that it used only binocular VESPA. Recording VESPA with monocular stimulation would allow to determine whether the VESPA method can account for visual dysfunction caused by acute or a previous occurrence of unilateral optic neuritis. Furthermore, a number of participants (13 MS, 12 controls) were excluded from the analysis due to noisy data. However, in clinical setting a reduced set of electrodes is used and thus more care is taken to ensure the top quality of data from each channel compared to the present high-density EEG set-up. In addition, the longitudinal analysis could be enhanced through larger sample sizes in the follow-up years.

The results obtained exhibited P100-like VESPA component latencies which were much later than anticipated and typically presented in the literature [15]. However, this feature is consistent across all responses for all years and is most likely a result of the testing procedure or the set up. As a consequence, this widespread prolongation in P100-like VESPA component did not affect the results of this study.

### Future directions

Our findings indicate that the VESPA method has promise to be a reliable and reproducible method that can adequately discriminate between MS patients and healthy controls. Although the findings of the present preliminary and exploratory study are promising, future studies are needed to determine whether the VESPA has utility in clinical practice as an efficient method to evaluate visual function, to be used as a prognostic marker and to monitor MS disease progression. The objective of utilizing VESPA in clinical practice is conceivable as VEPs are already commonly acquired in clinics, and the recording procedures of VEP and VESPA methods are similar. Furthermore, the acquisition and analysis of VESPA is fast with the high computational power of modern computers, especially if the pre-processing and analysis steps are standardized and automatized. The future studies could compare P100-like VESPA activations to findings from imaging techniques such as retinal nerve fiber layer imaging (e.g. optical coherence tomography), and optic nerve magnetic resonance imaging, which may provide further advances in understanding the pathophysiological mechanisms in MS and related visual dysfunction, such as optic neuritis. These future studies could elucidate neural mechanisms of visual dysfunction in MS and investigate the potential clinical utility of VESPA method as an efficient method for detecting and monitoring visual dysfunction in MS.

## Supporting Information

S1 Dataset Demographical, behavioural and electrophysiological data of the participants. (XLSX)

S1 Table The sensitivity and specificity of P100-like VESPA latency at Month 0 at various cut-off levels. (DOCX)

## Abbreviations

EDSS: Kurtzke Expanded Disability Status Scale
EEG: electroencephalography
MS: multiple sclerosis
ON: optic neuritis
RRMS: relapsing-remitting multiple sclerosis
SPMS: secondary-progressive multiple sclerosis
VEP: visual evoked potential
VESPA: Visual Evoked Spread Spectrum Analysis
