# Supplementary material for: Delayed P100-Like Latencies in Multiple Sclerosis: A Preliminary Investigation Using Visual Evoked Spread Spectrum Analysis
Source: PLoS One. 2016 Jan 4;11(1):e0146084. doi: 10.1371/journal.pone.0146084 (PMC4699709; doi:10.1371/journal.pone.0146084)
Supplement: S1 Table — (DOCX) [file pone.0146084.s002.docx]

| **P100-like VESPA latency cut-off** | Sensitivity | 1 - Specificity |
| --- | --- | --- |
| 95.48 | 1.000 | 1.000 |
| 108.24 | .977 | 1.000 |
| 121.00 | .977 | .976 |
| 122.95 | .977 | .881 |
| 124.90 | .977 | .857 |
| 126.85 | .930 | .833 |
| 128.80 | .930 | .810 |
| 130.80 | .930 | .762 |
| 132.25 | .930 | .738 |
| 133.20 | .930 | .714 |
| 134.70 | .907 | .714 |
| 136.65 | .884 | .714 |
| 139.10 | .860 | .714 |
| 142.05 | .837 | .714 |
| 145.50 | .814 | .667 |
| 149.45 | .791 | .643 |
| 153.35 | .767 | .643 |
| 157.25 | .767 | .619 |
| 160.20 | .744 | .619 |
| 162.65 | .744 | .571 |
| 165.60 | .744 | .524 |
| 168.05 | .651 | .500 |
| 170.00 | .651 | .452 |
| 171.95 | .628 | .357 |
| 173.90 | .581 | .310 |
| 175.90 | .581 | .262 |
| 177.85 | .558 | .214 |
| 179.80 | .535 | .190 |
| 181.75 | .512 | .167 |
| 183.20 | .512 | .143 |
| 184.20 | .488 | .143 |
| 185.70 | .442 | .143 |
| 187.65 | .442 | .119 |
| 189.10 | .395 | .119 |
| 191.05 | .372 | .119 |
| 196.45 | .349 | .095 |
| 201.35 | .349 | .071 |
| 203.30 | .326 | .071 |
| 205.65 | .302 | .071 |
| 207.60 | .302 | .048 |
| 209.20 | .302 | .024 |
| 214.10 | .279 | .024 |
| 219.95 | .209 | .024 |
| 224.40 | .186 | .024 |
| 230.30 | .163 | .024 |
| 237.60 | .163 | 0.000 |
| 242.50 | .140 | 0.000 |
| 246.45 | .093 | 0.000 |
| 256.25 | .070 | 0.000 |
| 265.05 | .047 | 0.000 |
| 282.70 | .023 | 0.000 |
| 299.40 | 0.000 | 0.000 |

Note. MS is coded as a positive actual state of the measure.
